# Into the unknown: a literature review of helminths of amphibians and reptiles in Mesoamerica

**DOI:** 10.1186/s13071-026-07579-8

**Published:** 2026-07-21

**Authors:** Tiffany Estrada-Pull, Eduardo Boza-Oviedo, Paula Alfaro-Segura, Jairo Alfonso Mendoza-Roldan, Alberto Solano-Barquero, Domenico Otranto, Alicia Rojas

**Affiliations:** 1 Laboratory of Helminthology, Faculty of Microbiology, University of Costa Rica, San José, Costa Rica; 2 Escuela de Medicina Veterinaria, Universidad Nacional, Heredia, Costa Rica; 3 Proyecto Ixchel (Centroamérica), San José, Costa Rica; 4 Centro de Investigación en Enfermedades Tropicales, University of Costa Rica, San José, Costa Rica; 5 Department of Veterinary Medicine, University of Bari, Bari, Italy; 6 Department of Veterinary Clinical Sciences, City University of Hong Kong, Hong Kong, China

**Keywords:** Helminths, Herpetofauna, Mexico, Guatemala, Belize, Honduras, El Salvador, Nicaragua, Costa Rica, Panama

## Abstract

**Background:**

Helminths are an important but understudied component of amphibian and reptile biodiversity, particularly in Mesoamerica, a global biodiversity and cultural hotspot.

**Methods:**

We conducted a comprehensive literature review of helminth parasites reported from amphibians and reptiles across Mesoamerica, yielding 71 publications with 1195 specimen records. Data extracted from each article included geographical location of the report, co-author affiliation and country of origin, host and parasite taxonomic details, deposition in taxonomic collections, and pathological data. In addition, a host–parasite association network was built to depict the most studied relationships.

**Results:**

This scoping review analyzed publications on helminths in Mesoamerican amphibians and reptiles, revealing clear geographic, taxonomic, and research biases. Most studies were published after 2000, peaking between 2006 and 2010, and focused largely on host–parasite relationships and species descriptions, likely reflecting the early consolidation of parasitological research in the region and the need to establish baseline biodiversity data. Research was concentrated in Costa Rica and Mexico, with Costa Rica contributing nearly half of all studies, and countries such as Honduras and El Salvador with no reports at all. However, much of the work was led by foreign, especially United States, institutions, highlighting the dominance of exogenous research efforts. Helminth diversity was dominated by nematodes (64.1%), followed by platyhelminths and acanthocephalans. A small number of parasite species and host species accounted for a disproportionately large share of records, particularly amphibians like *Rhinella horribilis* and *Leptodactylus melanonotus*, as well as *Anolis* lizards. Network analysis showed that these hosts act as key hubs supporting diverse parasite communities. Most parasite specimens were deposited in North American collections rather than local institutions, especially in the United States. Additionally, studies rarely included ecological or pathological data, focusing mainly on descriptive taxonomy. Overall, the findings highlight strong sampling biases, limited regional research capacity, and a need for more integrative ecological and pathological approaches in Mesoamerican parasitology.

**Conclusions:**

Our review underscores significant gaps in current knowledge, advocating for increased endogenous research and genuine international cooperation, systematic sampling of understudied host groups, and integration of One Health approaches to enhance understanding of host–parasite dynamics, biodiversity conservation, and potential zoonotic risks in Mesoamerica.

**Graphical Abstract:**

**Figure:**
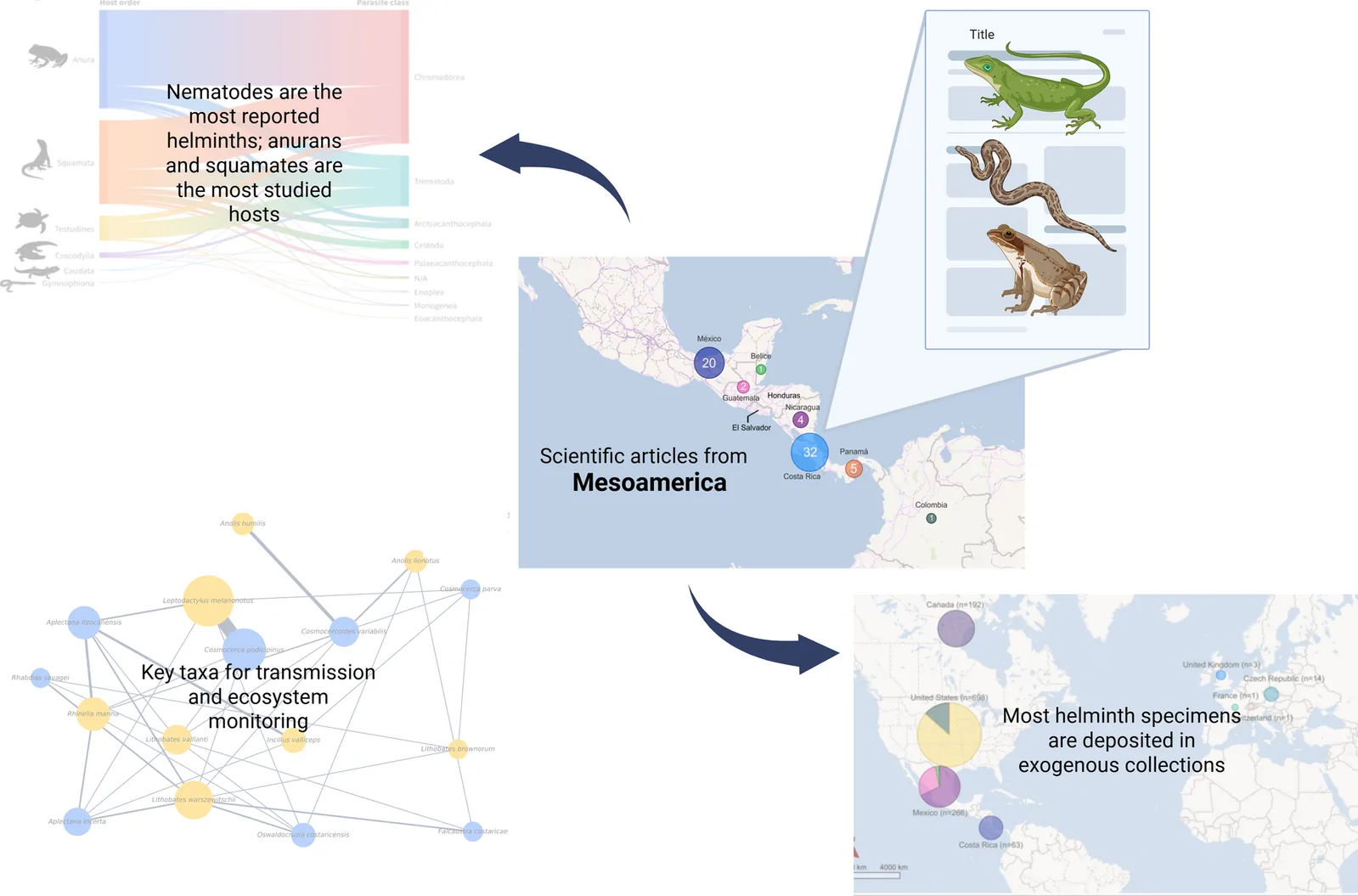

**Supplementary Information:**

The online version contains supplementary material available at https://doi.org/10.1186/s13071-026-07579-8.

## Background

Amphibians and reptiles, collectively known as herpetofauna, are essential components of terrestrial and freshwater ecosystems, contributing to a wide range of ecological functions such as nutrient cycling, energy flow through predator–prey trophic webs, seed dispersal, pollination and bioturbation [1]. Despite their ecological importance, herpetofauna has been less studied than mammals and birds, as reflected by their disproportionately low representation in published literature [2]. In addition, many amphibian and reptile populations in Mesoamerica and worldwide have become increasingly vulnerable to extinction, due to habitat deforestation, water pollution, illegal pet trade and emerging infectious diseases like chytridiomycosis and snake fungal disease. Amphibians of the genus *Atelopus*, including the Panamanian golden frog (*Atelopus zeteki* Dunn, 1933), represent emblematic examples of these widespread declines [3–6]. Therefore, the rapid decrease in herpetofauna populations has led researchers to prioritize understanding the threats they face in order to support conservation efforts [7].

Helminths, including cestodes, trematodes and nematodes, are a diverse group of parasitic organisms, grouped primarily by their worm-like morphology and their complex life cycles [8]. These parasites may play a significant role in the health of both amphibians and reptiles, mainly when combined with other stressors (e.g., climate change) and ecological disturbances (e.g., population fragmentation and invasive species). They can increase host vulnerability to predation, disease and reduced reproductive success, indirectly contributing to population declines [9]. For instance, Pacific tree frogs (*Pseudacris regilla* Baird and Girard, 1852) infected with trematodes of the genus *Ribeiroia* often develop limb abnormalities that hinder their mobility, reducing their ability to avoid predators and ultimately increasing mortality, which can negatively impact reproduction efforts [7, 10].

Beyond their effects at the individual host level, parasites are increasingly recognized as key components of ecological systems [7]. For example, parasite diversity within a host can reflect the diversity of intermediate and definitive hosts involved in their life cycles, providing insight into trophic interactions. Additionally, parasites respond to environmental changes, and can serve as indicators of ecosystem health, biodiversity and food-web structure [11]. Moreover, understanding the geographic distribution of parasite species across different host groups is essential for elucidating host–parasite interactions and their ecological significance [12].

Overall, helminths may use herpetofauna as definitive hosts, such as *Oswaldocruzia costaricensis* Bursey and Goldberg, 2005 in frogs, *Mesocoelium monas* Rudolphi, 1819 toads, and lizards, and different species of *Kalicephalus* in snakes [10, 13, 14]. In contrast, several taxa of zoonotic parasites use amphibians and reptiles as intermediate or paratenic hosts [7]. For instance, *Spirometra* spp. may induce sparganosis in humans and intestinal infections in canids and felids [15]. This further highlights the relevance of animal hosts in the One Health framework [16, 17]. Interactions between humans and herpetofauna are context-dependent, as urbanization, habitat loss and fragmentation often lead to population declines of sensitive species rather than increased contact, while favoring a few tolerant or synanthropic species to adapt to peridomestic environments [18, 19]. On the other hand, consumption of amphibian and reptile meat products (e.g., consumption of crocodilian meat in Europe and North America), and pet trade have intensified interactions and increased potential zoonotic infections [19, 20].

In this review, Mesoamerica was delimited following the Mesoamerican Biological Corridor (MBC) framework, explained further in the Methods section, extending from Southern Mexico to Northern Colombia. This area occupies less than 0.5% of Earth's terrestrial surface, yet it is estimated to contain about 7–10% of its animal species [21]. This high biodiversity can be explained by its geological origins, high environmental diversity, significant variation in precipitation and nutrient-rich volcanic soil [22]. Additionally, migration of species from North and South America due to the formation of the Panamanian isthmus, known as the “Great American Biotic Exchange”, has led to a great diversity in its flora and fauna [23]. Because of this richness, endemism as well as the political and cultural characteristics common to this location, Mesoamerica is regarded as a geographical region with a focal point for biodiversity conservation efforts over the past decades [21, 22].

Despite the ecological significance of helminths in herpetofauna and the finding of these parasites in different geographical regions from the Americas, like Costa Rica [15], Mexico [24], and Colombia [25], existing knowledge in Mesoamerica remains fragmented and geographically dispersed. This lack of integration limits broader understanding of host–parasite relationships at a regional scale. Therefore, the aims of this scoping review are to systematically synthesize published information on helminth parasites of herpetofauna in Mesoamerica, with emphasis on host–parasite associations, parasite diversity, geographic distribution, and research trends. Additionally, this review identifies major knowledge gaps and discusses future directions to improve understanding of host–parasite interactions and their implications for ecology, conservation, and zoonotic risk.

## Methods

A comprehensive search was conducted to identify all available references on parasites of amphibians and reptiles in Mesoamerica. Databases such as PubMed (https://pubmed.ncbi.nlm.nih.gov/), Google Scholar (https://scholar.google.com/) and Scopus (https://www.scopus.com/) were used to retrieve relevant literature to this study. These databases were selected due to their broad coverage of biomedical, veterinary, ecological, and zoological literature relevant to parasitology and herpetology. The search terms included “Mesoamerica” and “Mesoamérica” along with the name of each country in the region including “México”, “Belize/Belice”, “Guatemala”, “El Salvador”, “Honduras”, “Nicaragua”, “Costa Rica”, “Panamá”, and “Colombia”, and combinations of the following keywords in English and Spanish: “parasites”, “helminths”, “nematodes”, “trematodes”, “cestodes”, “reptiles”, “amphibians”, and the scientific groups “Nematoda”, “Trematoda”, “Cestoda”, “Amphibia” and “Reptilia”. Keyword variations were also used to broaden the search coverage. Databases were last visited on January 9th, 2025.

For the purposes of this review, Mesoamerica was defined following the MBC framework [21, 26], extending from the Isthmus of Tehuantepec in Southern Mexico to the Urabá-Cupica area in Northern Colombia. We acknowledge that this delimitation is broader than the strict biogeographic definition of the Mesoamerican dominion proposed by Morrone in 2014 [27], which restricts the area to the lowlands of Southern and Central Mexico together with Guatemala, Belize, Honduras, El Salvador and northern Nicaragua, and excludes Costa Rica, Panama and The Chocó-Darién. We, instead adopted the MBC framework for two reasons: (i) it is grounded on ecological connectivity of lowland habitats—a more relevant criterion for parasite–host interactions than strict regionalization schemes—and (ii) the herpetofaunal communities of Lower Central America, the Darién and the northernmost Chocó share substantial faunistic affinities, supporting their treatment as a single ecologically continuous region for the purposes of host–parasite analyses [22, 28]. Politically, the region thus includes parts of Veracruz and Oaxaca, all of Tabasco, Campeche, Yucatán, Quintana Roo and Chiapas in Mexico, as well as Belize, Guatemala, Honduras, El Salvador, Nicaragua, Costa Rica, Panama, and portions of Chocó and Antioquia in Colombia. Only studies conducted within this geographic scope and those focused on helminth parasites of amphibians and reptiles were included. Studies not meeting these criteria, as well as records focusing on non-helminth parasites, were excluded. Studies containing records from more than one country were classified as “mixed” for geographic analyses and duplicates were removed prior to screening. For example, even though many articles were found for Mexico, many of them didn’t apply because they weren’t within the geographic scope of this study.

Taxonomic names of both hosts and parasites were verified using the World Register of Marine Species (WoRMS; https://www.marinespecies.org/), AmphibiaWeb (https://amphibiaweb.org/), The Reptile Database (https://reptile-database.reptarium.cz/) and the judgment of herpetologists to ensure the use of valid and up-to-date classifications. Accepted current nomenclature was used for standardization in the dataset, while historical names reported in the original publications were also retained to preserve traceability and consistency with the reviewed literature (e.g., Mesoamerican records originally attributed to *Rhinella marina* likely correspond to *R. horribilis* under current taxonomy) (Supplementary File 1). For parasite specimens deposited in collections, acronyms are as specified in Table 1.

**Table 1:** Acronyms and corresponding helminthological collections referenced in the reviewed literature on parasites of Mesoamerican amphibians and reptiles from 1957 to 2023

Acronyms	Name of institution/university	Country
USNPC	United States National Parasite Collection	USA
CNHE*	National Helminth Collection (Colección Nacional de Helmintos)	Mexico
CHCR	Helminthological Collection, Laboratory of Helminthology, Faculty of Microbiology, University of Costa Rica	Costa Rica
HWML	Harold W. Manter Laboratory of Parasitology, University of Nebraska—Lincoln	USA
COPAFRO	Parasite Collection, El Colegio de la Frontera Sur, Chetumal Unit	Mexico
IPCAS	Institute of Parasitology, Academy of Sciences of the Czech Republic	Czech Republic
DZUT	Department of Zoology, University of Toronto	Canada
MHNG	Geneva Natural History Museum, Invertebrate Collection (INVE)	Switzerland
CHIB-UNAM*	Helminthological Collection, Institute of Biology, National Autonomous University of Mexico (UNAM)	Mexico
BMNH	Natural History Museum, London	United Kingdom
ECOPA	Colección de Parásitos, ECOSUR-Chetumal	Mexico
MNHNP	Muséum National d'histoire Naturelle Paris, France	France
UMMZ	Museum of Zoology, University of Michigan, Ann Arbor, Michigan	USA

^*^The CNHE (National Helminth Collection) and CHIB-UNAM (Helminthological Collection of the Institute of Biology, UNAM) are distinct collections housed at the National Autonomous University of Mexico

A database was assembled with 33 features, that included taxonomic classifications of both hosts and parasites, geographical location of the report, infection information such as prevalence, anatomical location of the parasites and pathology (Supplementary Table 1). To account for the numerous taxonomic changes in amphibians, reptiles, and the parasite groups studied here, we carefully cross-checked original records of localities and names to deliver an updated taxonomic framework whenever possible. Additional fields included voucher specimen information and deposition repositories (collections) for hosts and parasites, as well as author affiliations and country of the affiliation. Author affiliations reported in each publication were categorized according to the geographic location of the affiliated institution. Affiliations corresponding to institutions located within Mesoamerica were considered endogenous, whereas affiliations outside the region were categorized as exogenous. Classification was based exclusively on institutional affiliation and not on author nationality. Moreover, each author’s affiliation from each article was registered and counted for descriptive analysis of endogenous or exogenous contributions. Descriptive statistics graphs were built using Google Sheets and Flourish, and maps showing distribution of generated research in Mesoamerica as well as the Helminthological Collections where material has been deposited were created in PowerBI (Microsoft^®^).

Host–parasite associations were compiled from the curated database provided in this study. Records lacking host or parasite identification were removed, as were entries corresponding to non-specific parasite identifications. Each unique host–parasite pair was retained, and repeated associations were quantified to allow edge weighting. Then, a bipartite host–parasite network was constructed, in which hosts and parasites were treated as distinct node types. Edges represented documented associations between hosts and parasite species. Edge weights corresponded to the frequency of each host–parasite interaction in the dataset.

Additionally, weighted degree (sum of edge weights per node) was calculated for all nodes, and the network was filtered to retain the top 20 nodes with the highest weighted degree, highlighting the most connected hosts and parasites. Host or parasite nodes in the network were retained when these related to other top 20 nodes. Therefore, highly connected nodes, but to other species not present in the ranking were filtered out. The network was generated using Python with the NetworkX library and visualized with Matplotlib. Node size was scaled proportionally to weighted degree to reflect connectivity. Hosts and parasites were assigned distinct colors for visual differentiation, and edges were drawn with widths proportional to their weights. A force-directed spring layout was applied, with increased spacing parameters to reduce node overlap and improve readability.

## Results

### Overview of the analyzed literature

**Fig. 1:**
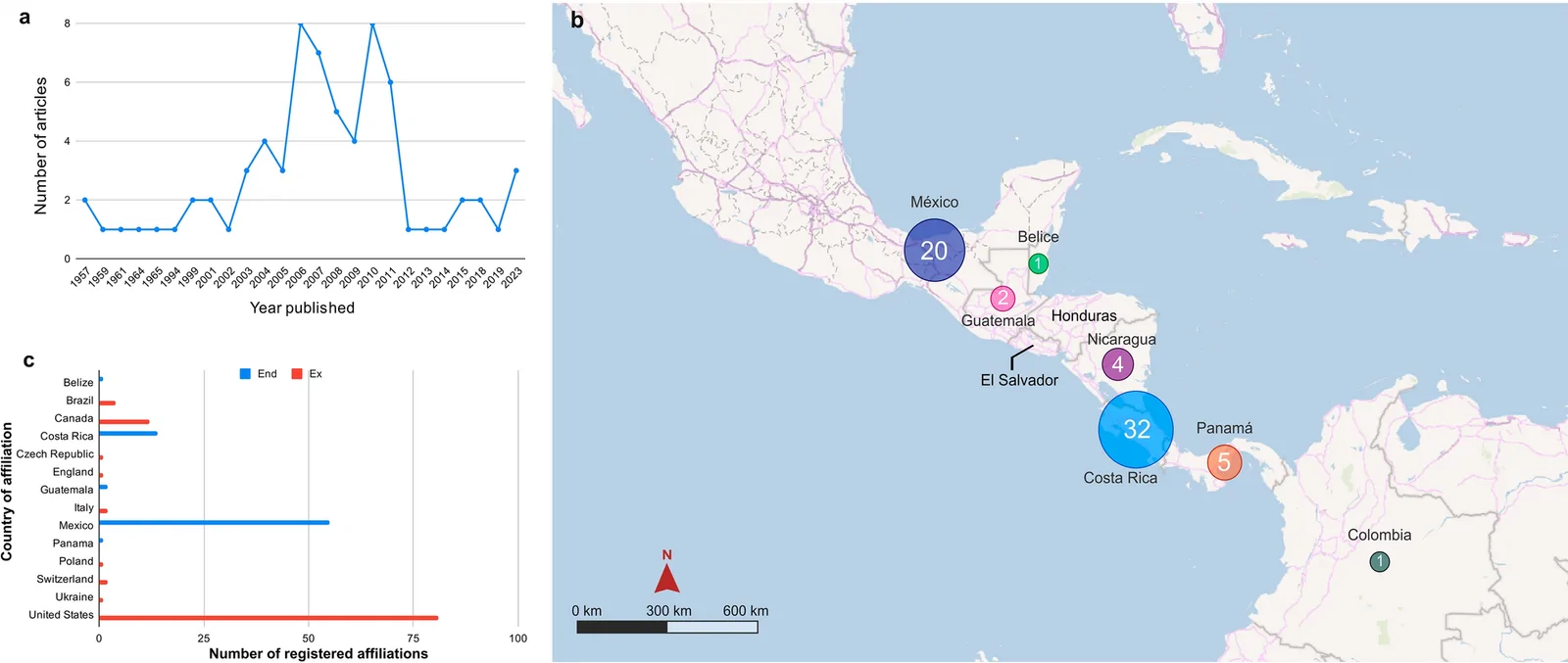
Historic overview of reports about helminths in amphibians and reptiles in Mesoamerica mined from a bibliographic revision. **a** Number of published records per year from 1957 to 2023. **b** Number of publications per country. **c** Author affiliations reflecting endogenous vs exogenous research

A total of 131 records of publications were identified through database searches, of which 71 met the inclusion criteria and were included in the final dataset. These included 66 peer-reviewed journal articles, one book, and four thesis dissertations. Of these, 63 were written in English and eight in Spanish. Of these 71 publications, 1195 host–parasite records were obtained, 554 corresponding to helminths in amphibians, and 641 to helminths in reptiles. The reviewed literature spanned from 1957 to 2023. The temporal distribution of articles (Fig. 1a) showed almost a complete absence of publications before the 2000s, followed by a gradual increase and a marked peak between 2006 and 2010. After 2011, the number of articles decreased, with only a few published in recent years. The studies published during this period mainly consisted of specific host–parasite associations, including the description of new species and characterizations of parasite taxa.

Publications were concentrated in Mexico, Costa Rica, Panama and Nicaragua (Fig. 1b). Out of the 71 publications included in this review, Costa Rica contributed the largest share (32/71, 45.1%). Costa Rica’s output was influenced by intensive studies conducted in the Área de Conservación de Guanacaste located in the North Pacific (9/32, 28.1%). Few studies were reported from Belize, Colombia and Guatemala and no articles were found in Honduras and El Salvador. In addition, six studies were “mixed” (records from Panama, Costa Rica, Nicaragua and Guatemala). In Costa Rica and Mexico, most publications focused on documenting helminth fauna in a wide variety of reptile and amphibian hosts, including reports of new species (29 out of 71 articles), new host and locality records, and further characterization of regional helminth biodiversity.

Analysis of author affiliations revealed a predominance of exogenous research over endogenous contributions (Fig. 1c). A total of 178 institutional affiliations were recorded. Endogenous contributions were mainly from Mexico (30.9%) and Costa Rica (7.9%), with contributions from Belize (0.56%), Guatemala (1.1%) and Panama (0.56%). Exogenous research was primarily conducted in different institutions in the United States (USA), which was the most dominant contributor (45.5%), Canada (6.7%), Brazil (2.2%) and several European countries (e.g., England, Poland, Switzerland, Italy, Ukraine) (4.5%).

### Parasite records according to their taxonomical categories

**Fig. 2:**
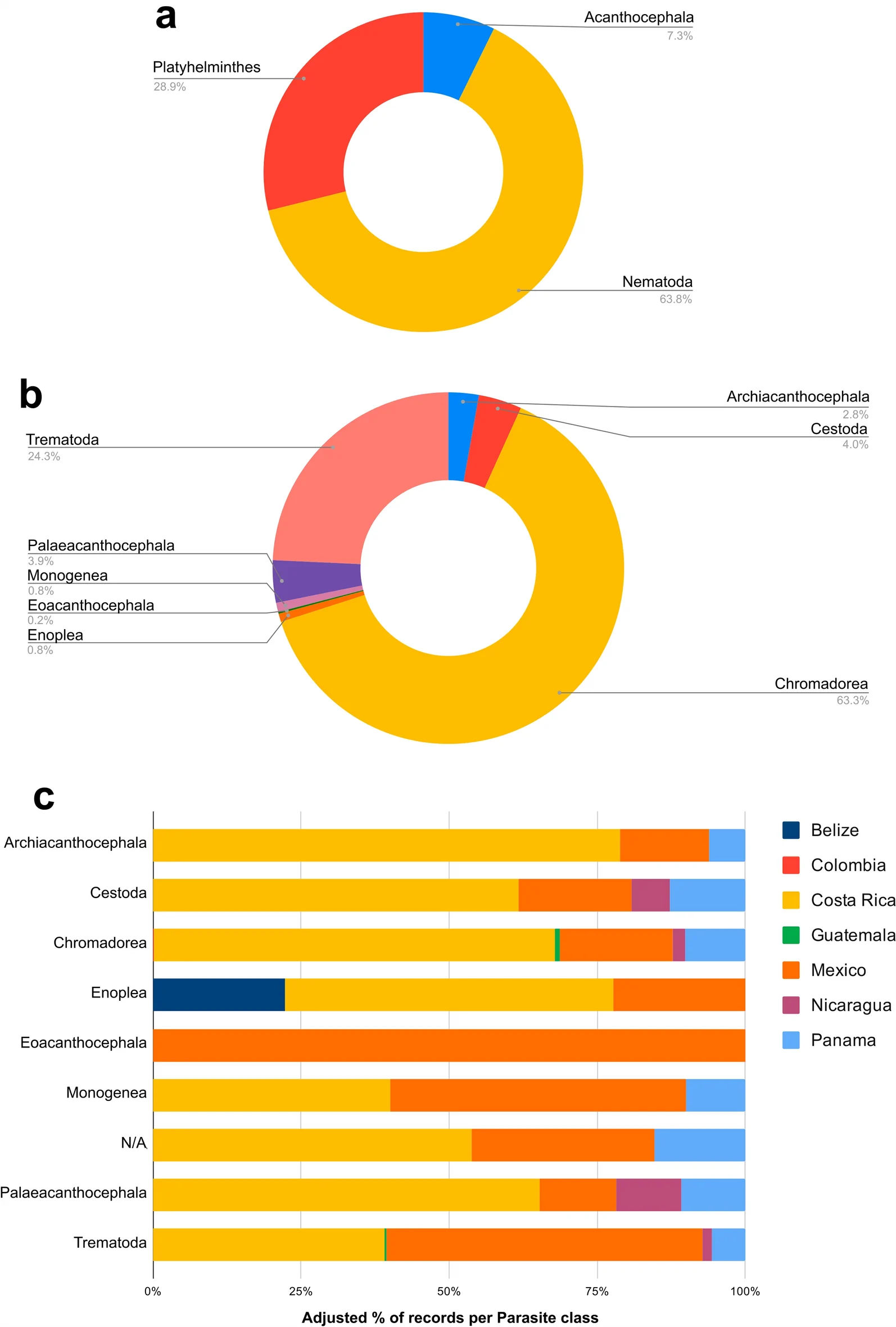
Taxonomic diversity of helminths reported in Mesoamerica. **a** Distribution of records by Phyla (%), **b** class (%) and **c** parasite class by country

Most helminth records across Mesoamerica corresponded to the phylum Nematoda (63.8%), followed by Platyhelminthes (28.9%) and Acanthocephala (7.3%) (Fig. 2a). A more detailed breakdown by class in those records with complete taxonomic classification (Fig. 2b) showed that the largest proportion of hits belonged to Chromadorea (63.3%), followed by Trematoda (24.3%), Cestoda (4.0%) and Palaeacanthocephala (3.9%). Other classes, such as Archiacanthocephala, Monogenea, Enoplea and Eoacanthocephala, are represented by fewer records: 4.6% summed together. In addition, the distribution of parasite class among Mesoamerican countries (Fig. 2c) showed a clear geographic bias in helminth reports, with Costa Rica and Mexico contributing to the largest proportion of records and diversity of helminth classes. In contrast, Colombia, Belize and Guatemala displayed much fewer results.

### Parasite records according to their hosts

**Fig. 3:**
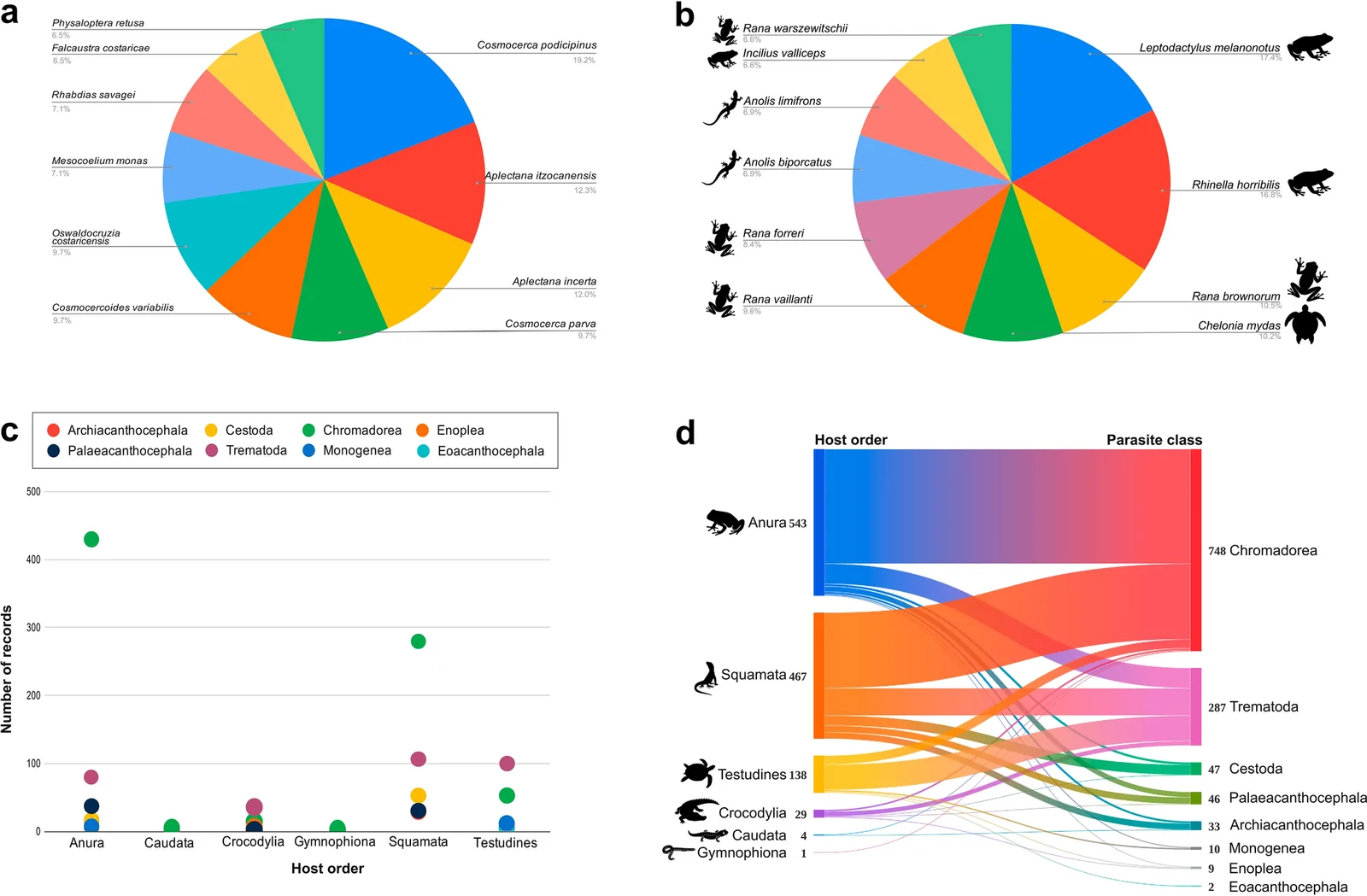
Helminth and acanthocephalan worms recorded from amphibian and reptile species across Mesoamerica. **a** Top 10 parasite species and their percentage in the analyzed articles, book chapters and dissertations. **b** Top 11 host species (%) for which parasites were recorded. **c** Parasite class diversity across different host orders. **d** Connections between parasite classes and host orders

The dataset compiled 276 distinct helminth taxa represented across the 1195 host–parasite association records. The ten most frequently recorded parasite species accounted for 25.8% (308/1195) of all records. The analysis of hosts associated with each recorded parasite species revealed several patterns. Within this top 10 subset, *Cosmocerca podicipinus* Baker and Vaucher, 1984 (19.2%), *Aplectana itzocanensis* Bravo Hollis, 1943 (12.3%) and *Aplectana incerta* Caballero, 1949 (12.0%) were the most frequently recorded helminth parasites, followed by the nematodes *Cosmocercoides variabilis* (Harwood, 1932), *Cosmocerca parva* Travassos, 1925, and *Oswaldocruzia costaricensis* Bursey and Goldberg, 2005, each reaching 9.7% of the records within the subset. The remaining four species (*Rhabdias savagei* Bursey and Goldberg, 2005; *Mesocoelium monas* Rudolphi, 1819 Teixeira de Freitas, 1958; *Falcaustra costaricae* Bursey, Goldberg and Miller, 2004; and *Physaloptera retusa* Rudolphi, 1819) contributed 27.2% collectively within the top-10 parasite records (Fig. 3a).

On the other hand, a total of 203 unique herpetofauna species were identified in the dataset. The top 10 host species (Fig. 3b) accounted for 27.9% (333/1195) of the total records. Among these, 17.4% corresponded to the amphibians *Leptodactylus melanonotus* Hallowell, 1861, followed by 16.8% for *Rhinella horribilis* Wiegmann, 1833, and 10.5% for *Rana brownorum* Baird, 1859. Two species of *Anolis* lizards (*Anolis biporcatus* Wiegmann, 1834 and *Anolis limifrons* Cope, 1863) collectively represented 13.8% of the subset records. The remaining hosts included other anuran species (*Rana vaillanti* Brocchi, 1877, *Rana forreri* Boulenger, 1883, *Rana warszewitschii* Schmidt, 1857, and *Incilius valliceps* Wiegmann, 1833) and a single turtle species, *Chelonia mydas* Linnaeus, 1758, representing 41.4% summed together.

Overall, a small number of host species accounted for a large proportion of the recorded parasite associations in the dataset. Moreover, the distribution of parasite classes differed among host orders (Fig. 3c). The orders Squamata and Anura exhibited the highest diversity of parasite classes, with representatives from nearly all of those recorded in the study. Chromadorea and Trematoda were the most frequently reported parasite classes, while Eoacanthocephala and Monogenea appeared less frequently and were restricted to a few host orders. Alternatively, Crocodylia, Caudata and Gymnophiona were represented by comparatively fewer parasite records and lower reported parasite diversity. Regarding the connections between parasite classes and their corresponding host orders (Fig. 3d), a clear overlap was observed among several host groups showing shared parasite taxa. As mentioned above, Anura and Squamata displayed the highest number of associations (543 and 467 connections, respectively), involving multiple parasite classes, including Chromadorea, Trematoda, and Archiacanthocephala. Testudines showed 138 associations, primarily with Trematoda and Chromadorea, while Crocodylia (29) were mainly associated with Trematoda, Cestoda, and Acanthocephala. Caudata exhibited a smaller number of associations (4), mainly with Archiacanthocephala and Chromadorea, whereas Gymnophiona presented the fewest connections overall (1).

**Fig. 4:**
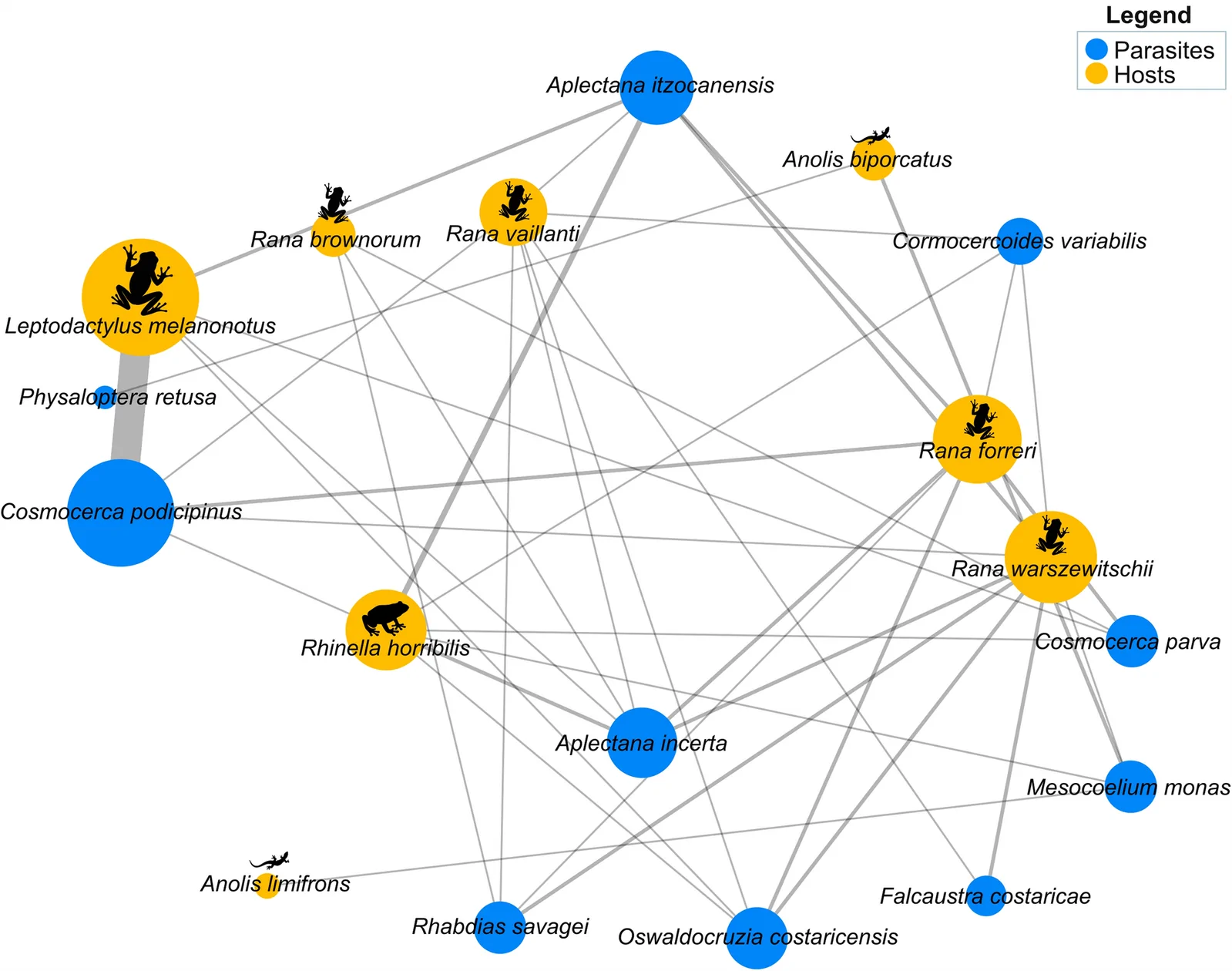
Host–parasite association network. Nodes represent host (orange) and parasite (blue) taxa, with node size proportional to weighted degree. Edges indicate interactions, with thickness reflecting frequency. The network shows 16 of the 20 highest weighted-degree nodes; the remaining four were removed because they had no edges with other top-ranked nodes

The weighted bipartite network revealed a heterogeneous structure dominated by a smaller number of highly connected taxa (Fig. 4). After filtering, the top 20 nodes ranked by weighted degree represented the core of the interaction network and included both hosts and parasites with disproportionately high connectivity. Among hosts, *R. horribilis* (*n* = 520), *L. melanonotus* (*n* = 40), *R. vaillanti* (*n* = 26), *R. brownorum* (*n* = 23), *R. forreri* (*n* = 23), *A. biporcatus* (*n* = 22), *R. warszewitschii* (*n* = 21), and *A. limifrons* (*n* = 19) (Supplementary File 1), formed central hubs supporting multiple parasite species, reflecting a high number of recorded host–parasite associations in the dataset. Other hosts such as *C. mydas* (*n* = 34), and *Dermatemys mawii* Gray, 1847 (*n* = 18), were highly connected to a variety of parasites but not included in the network since their associated parasites were not among the top 20. Parasite hubs were primarily represented by nematodes, with *C. podicipinus* (*n* = 59) exhibiting the highest weighted degree, followed by *A. itzocanensis* (*n* = 37), *A. incerta* (*n* = 37), *C. variabilis* (*n* = 30), *C. parva* (*n* = 30), *O. costaricensis* (*n* = 30), *Mesocoelium monas* (*n* = 22), *Rhabdias savagei* Bursey and Goldberg, 2005 (*n* = 22), *Falcaustra costaricae* Bursey, Goldberg and Miller, 2004 (*n* = 20) and *Physaloptera retusa* (*n* = 19). These parasites showed repeated associations across multiple hosts and accounted for a large proportion of the recorded host–parasite interactions.

Edge weighting revealed recurrent host–parasite interactions, emphasizing non-random association patterns. Several hosts, particularly *R. horribilis* and *L. melanonotus*, supported diverse parasite assemblages, while certain parasites, notably *C. podicipinus*, connected multiple amphibian and reptile hosts, contributing substantially to overall network cohesion. Importantly, *L. melanonotus* frog and *C. podicipinus* were the most connected host–parasite nodes according to their weighted degree. Visualization of the spring-layout network highlighted clear clustering around highly connected hosts, with groups of parasite species aggregating on the same hosts, consistent with structured parasite communities rather than isolated infections. The marked variation in node size further demonstrated substantial differences in interaction intensity among taxa.

### Geographic distribution of research and biological collections

**Fig. 5:**
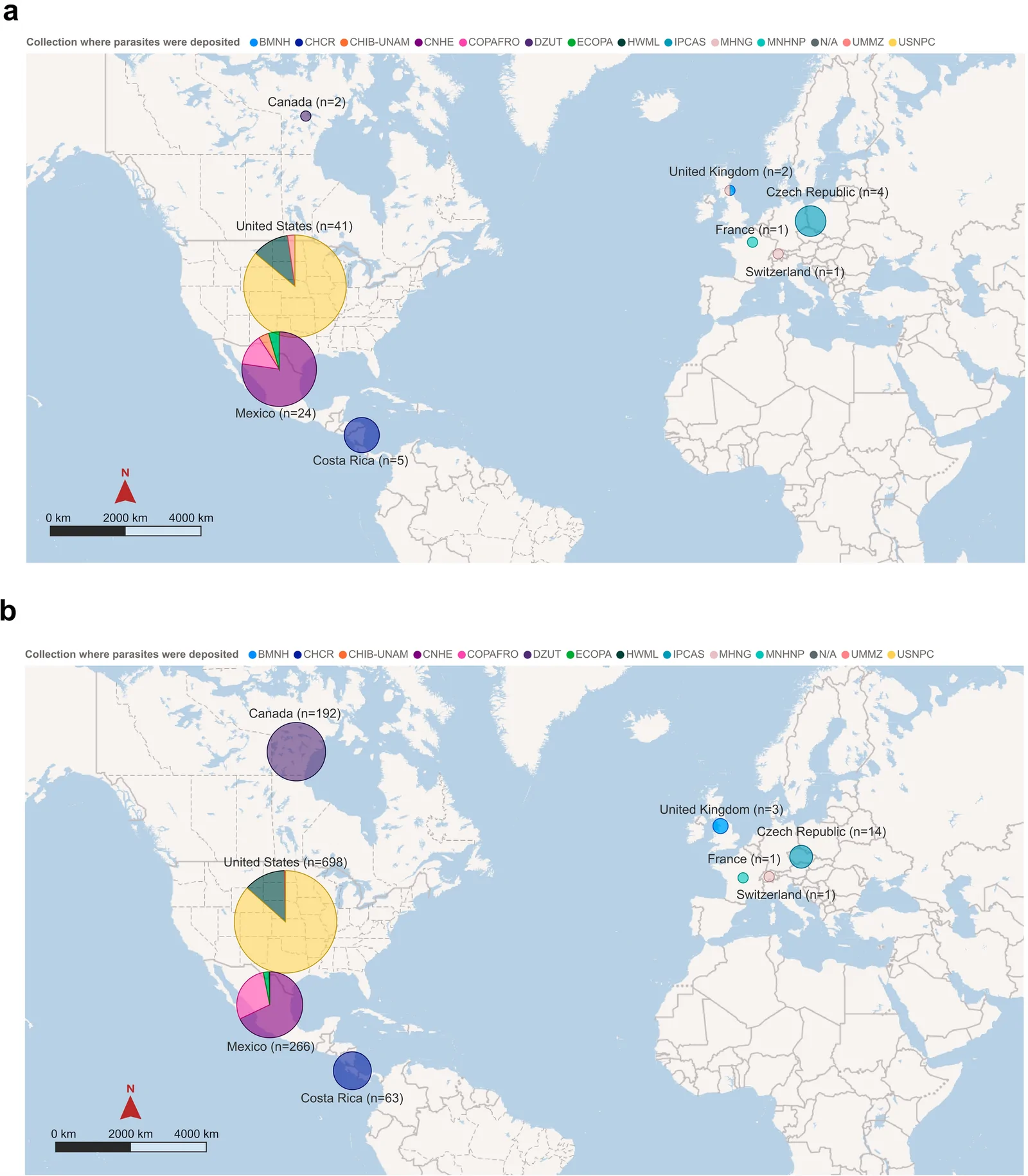
Map of institutions housing parasite collections. **a** Articles with explicit specimen deposition in a natural collection. **b** Number of specimens deposited in collections around the world

Most parasite specimens examined in this study were housed in a few major collections (Fig. 5a). Of the 71 articles reviewed, 62 (87.3%) reported deposition of parasite specimens in biological collections, some of them depositing the same species in different collections. The largest number of records was held by the USNPC (United States National Parasite Collection) (59.7%). Other notable collections were CNHE (Colección Nacional de Helmintos) in Mexico (30.6%), HWML (Harold W. Manter Laboratory) in the USA (8.1%) and CHCR (Colección Helmintológica del Laboratorio de Helmintología de la Facultad de Microbiología, UCR) in Costa Rica (8.1%). At the level of individual records, a similar pattern was observed (Fig. 5b). The majority of parasite specimens were concentrated in a few key collections, particularly the USNPC of the USA (603 records), followed by DZUT in Canada (192) and CNHE in Mexico (174). Additional substantial holdings were found in HWML in the USA (90), COPAFRO in Mexico (74), and CHCR in Costa Rica (63); the remaining collections each accounted for comparatively few records (≤ 14). Overall, parasite specimens were predominantly curated in North American collections (USA, Mexico, and Canada), with smaller representation in Costa Rican and European institutions (e.g., BMNH, IPCAS, MHNG, MNHNP). Moreover, 71 specimens were not deposited in a collection, or this information was not explicit in the source article. These parasites were mostly from Costa Rica (52), followed by Mexico (10), Guatemala (5), Belize (2), Panama (1) and Colombia (1).

Specimens were mostly deposited in exogenous collections, except of the case of Mexico, in which 328 specimens were reported in the analyzed time range, and 81.1% (266/328) of these worms were deposited in Mexican collections, and the rest in USA or UK collections. On the other hand, only 8.9% (64/721) of the specimens reported in Costa Rica were deposited in collections from this country, and 83.5% (602/721) of the reported Costa Rican specimens were found mainly in the USA. In addition, the totality of Nicaraguan (29) and Panamanian (52) records were deposited in the USNPC from the USA, and a similar situation was observed with Guatemala where two of the reported specimens were deposited in the USNPC from the USA and the other five records were not deposited at all. Overall, parasite specimens were predominantly curated in North American collections, with smaller representation in Costa Rican and European (England, Czech Republic, Switzerland, France) institutions.

### Anatomical, pathological and ecological information

Although not represented in the figures above, helminths were predominantly reported in the gastrointestinal system across most studies that specified anatomical localization. In contrast, very few studies included information on parasite-associated pathology, and in most cases, pathological findings were not reported. This lack of information makes it difficult to determine whether pathological effects were absent or simply not assessed or documented. Only one study by Santoro and colleagues [29] reported associated pathology, in the hawksbill turtle (*Eretmochelys imbricata* Linnaeus, 1766)*,* describing nodular lesions on the intestinal serosa and granulomatous lesions in the mucosa, submucosa, and muscular layers of the stomach and intestine, as well as in the liver and gallbladder. Similar to the limited reporting of pathological findings, ecological information was also largely absent from most records with only a small proportion (3/71, 4.2%) including ecological context (e.g., habitat characteristics, environmental conditions). This suggests that most studies have focused primarily on descriptive parasitology, with limited incorporation of ecological perspectives.

## Discussion

The present review exposes a pronounced geographical and temporal bias in helminth research conducted on amphibians and reptiles in Mesoamerica, with most studies concentrated in Mexico and Costa Rica, as well as little to no information available for Honduras, El Salvador, Belize and regions of Colombia. This pattern reflects broader trends in biodiversity research, where countries with stronger academic infrastructure, organization and long-standing herpetological programs contribute disproportionately to the available literature and specimens deposited in helminth collections. The scarcity of studies in other parts of the region likely results from combinations of at least some of the following aspects: insufficient funding, inadequate infrastructure and expertise for data collection, difficulties in collecting and publishing data, inaccessibility to research sites due to political conflicts or inappropriate use of research funds [30]. Future efforts should prioritize systematic surveys in underrepresented regions, where current records remain extremely limited.

Regarding the temporal publication trends, the increase in publications during the early 2000s likely reflects a period dominated by descriptive parasitology, focused on species inventories and new host and locality records. In line with broader trends in ecology, this period also marked the beginning of a shift toward incorporating the effects of environmental change on host–parasite interactions [31]. The subsequent decline in publications may be associated with shifts in research priorities, including a growing emphasis on molecular approaches and more interest in emerging infectious diseases, as well as fluctuations in scientific investment and political support for research in these countries [32].

Beyond geographic and temporal patterns, the distribution of author affiliations also reflects an important predominance of exogenous research. This asymmetry suggests research dependencies, in which biodiversity-rich countries with limited institutional capacity, training opportunities, and economic resources often provide biological material and field data, while analyses and publications are conducted abroad [33]. Previous studies have shown that national wealth is positively associated with data availability, as it influences investment in education and science, as well as the quantity and quality of scientific output [34]. Consequently, scientists in lower-income countries may face challenges, such as limited funding for in-depth analyses, which restrict their ability to generate, access, and disseminate biodiversity data [35]. However, economic factors alone do not explain these patterns. Other elements, including institutional organization, limited coordination and collaboration between international-local researchers, access to training opportunities, as well as misuse of funds play a critical role [36].

Additionally, language barriers may influence scientific participation, as English remains the dominant language of international communication, potentially limiting the visibility and dissemination of research [37]. However, this bias is not unidirectional. Recent evidence suggests that important biodiversity knowledge is published in non-English languages and is often overlooked by the global scientific community, leading to underrepresentation of existing data [38]. Studies suggest that scientific contributions published in non-English languages are often underrecognized, which can reduce the visibility of local research and limit its incorporation into study design, analysis, and broader scientific frameworks [39].

As a result, apparent knowledge gaps may in some cases reflect limitations in data accessibility and integration rather than true absence of information. Beyond the factors mentioned above, broader socio-political conditions may also influence patterns of research. Recent studies have shown that variables such as democratic access to information, freedom of expression, political stability, and levels of conflict can affect both the production and dissemination of biodiversity data across regions [40]. These factors may contribute to uneven reporting of species and parasite records, where research capacity is shaped by complex institutional and political contexts.

A central issue intertwined with the endogenous/exogenous patterns mentioned previously, is the persistence of the so-called scientific colonialism, parachute science or helicopter research in which scientists from foreign, higher-income institutions conduct fieldwork, collect and describe species, in a lower-income country with minimal or no involvement of local scientists, institutions or communities [41]. These practices lack academic inclusion and can delay new research opportunities and scientific development. These dynamics are rooted in colonial relationships that produced long-lasting disparities in access to resources, infrastructure and scientific opportunities [42]. Notably, linguistic and cultural barriers may further reinforce these patterns. The rapid loss of biodiversity occurring globally, including in developing countries, highlights the need for local biodiversity scientists who are indispensable for conservation efforts [35]. For this reason, we advocate for genuine collaborative research models that foster equitable partnerships and enable scientists from diverse regions and with diverse languages to combine their expertise toward shared scientific goals.

At the regional level, endogenous research was concentrated mostly in Costa Rica and Mexico, the two countries with the most developed national collections, such as the Colección Helmintológica de Costa Rica (CHCR) and the Colección Nacional de Helmintos (CNHE), respectively, as well as strong herpetological research programs in these regions. Similarly, other studies have observed that Mexico and Costa Rica keep the highest records of acanthocephalan diversity in Neotropical anurans [43]. This may reflect the fact that Mexico and Costa Rica, along with Guatemala, have the highest GDPs (according to the World Bank, 2025) in the region, which most likely facilitates greater investment in education, science, and biodiversity research. Moreover, Costa Rica allocates 0.34% of its GDP to research and development, and Mexico 0.26%. Although these are high numbers for the region, they remain modest compared to the OECD (Organization for Economic Co-operation and Development) average of 2.73%, which includes mainly high-income countries from North America, Europe, and the Asia–Pacific region, once again highlighting the gap in science investment between high-, middle- and low-income countries [44].

Conversely, the comparatively limited number of records from countries such as Colombia, Belize and Guatemala likely reflects differences in sampling effort, research focus and the availability of taxonomic expertise rather than true absences of these parasite groups. However, historical and socio-political factors play a significant role in shaping these patterns. In particular, periods of armed conflict, political instability, and varying levels of democratic governance across several Central American countries during the late twentieth century may have limited the development of scientific infrastructure and long-term biodiversity research programs. In contrast, countries such as Costa Rica benefited from relatively stable political conditions, sustained investment in education and research, and the establishment of key scientific institutions, which have collectively supported more consistent biodiversity documentation [40].

From a taxonomic perspective, helminth records from Mesoamerican amphibians and reptiles are dominated by nematodes, followed by platyhelminthes and lastly acanthocephalans. This pattern is consistent with findings from other regions, where nematodes typically represent the most frequently reported helminths in herpetofauna. Studies from South America [45, 46], Southeast and Western Asia [16, 47, 48], and Africa [49, 50] likewise document nematodes as the dominant helminth group in amphibians and reptiles. This suggests a global trend in their reported prevalence, which may reflect both biological patterns and methodological biases, including differences in detection, sampling, and taxonomic expertise [51].

However, these patterns may also be influenced by the taxonomic focus of researchers, potentially leading to underrepresentation of other helminth groups when not specifically targeted. Multi-host life cycles, seasonal host availability, rapid post-mortem deterioration and the need to use specialized techniques for identification in Platyhelminthes may hinder the estimation of true diversity [52]. Acanthocephala accounted for the least number of records, which is a similar pattern to global reports and from other regions in the tropics, where they tend to be infrequently reported due to their complex life cycles and host-specific ecology [43, 51]. Integrative approaches combining morphology and molecular tools will be necessary to clarify cryptic diversity and improve parasite identification [53].

Regarding the ten most frequently recorded parasite species in our dataset, the predominance of *A. itzocanensis* and *A. incerta* is consistent with patterns reported for neotropical amphibians and reptiles, being the most common genera found in herpetological hosts from the Neotropics [54]. They are cosmocercid nematodes of the digestive tract of amphibians and reptiles and typically exhibit low pathogenicity, often persisting in host populations because they have direct life cycles and can infect a broad range of amphibian and reptile species, which facilitates transmission and long-term maintenance in the ecosystem [55, 56]. Similarly, *C. parva* and *C. podicipinus* are cosmocercid nematodes widely reported in Neotropical frogs and are often generalists with minimal impact (i.e., low pathogenicity). *Cosmocercoides variabilis*, also among the top 10, belongs to a closely related but distinct cosmocercid genus and shares comparable ecological attributes. This also reflects the ease of detection and host abundance in these species [57]. However, *Cosmocerca* represents a taxonomically complex genus, and the broad distribution patterns reported for species such as *C. podicipinus* and *C. parva* highlight the need for further integrative taxonomic studies.

*Physaloptera retusa* is a common species in Neotropical lizards, has an indirect life cycle involving arthropod intermediate hosts, and is transmitted through trophic interactions [58]. In contrast, records of this parasite in amphibians [10, 59, 60] and reptiles [61–63] appear to be sporadic. In the Neotropics, they have been reported to act primarily as paratenic hosts for *Porrocaecum* spp., with birds as definitive hosts and earthworms as intermediate hosts [64]. Therefore, the occurrence of these parasites in herpetofauna likely represents incidental findings associated with trophic interactions rather than established host–parasite relationships.

*Rhabdias savagei*, *Mesocoelium monas* and *Falcaustra costaricae* are also ranked among the ten most frequently recorded species. *Rhabdias savagei* is a lungworm with a heterogonic life cycle that allows both free-living and parasitic generations, facilitating its persistence in humid neotropical environments [65]. *Mesocoelium monas* is a digenean trematode with an indirect life cycle commonly reported in the intestine of amphibians and reptiles across the Neotropics. On the other hand, *F. costaricae* is a nematode of the family Kathlaniidae associated primarily with anurans in Central America. The relatively high frequency of these three species in our dataset may partly reflect their broad host ranges and the geographic focus of the reviewed literature on areas where their respective host species are particularly abundant [65].

Species of *Oswaldocruzia* are parasites with a direct life cycle, commonly associated with amphibians and reptiles in the Neotropics. Records of *O. costaricensis*, included in the top 10 recorded parasite species, are derived from a small number of studies [65–68]. This may overrepresent its frequency and suggest that the prominence among the most recorded taxa reflects targeted sampling efforts rather than widespread occurrence in hosts and/or regions.

Beyond the diversity patterns, some of the recorded helminths also have important zoonotic implications. The genus *Mesocestoides* includes species with recognized zoonotic potential, although infections are rare and their transmission is not fully understood. Amphibians and reptiles are known to act as second intermediate hosts. Sporadic human infections have been reported (i.e., non-specific gastrointestinal symptoms), with possible exposure due to the consumption of raw snake meat and viscera containing tetrathyridia larval stages, contact with exotic pets (e.g., lizards), and snapping turtle [69]. In several Neotropical regions, amphibians and reptiles are also consumed as bushmeat, including iguanas, large frogs and caimans, which may further increase exposure to foodborne parasitic infections in certain cultural contexts [70, 71]. The detection of these parasites in this review underscores the ecological role that herpetofauna can play in maintaining complex yet poorly understood transmission cycles, reinforcing the importance of monitoring these parasites within a One Health framework.

Although recorded only once in our dataset [68], a record of encysted plerocercoids in *R. warszewitschii* in Costa Rica is noteworthy, as amphibians and reptiles can act as intermediate or paratenic hosts for zoonotic diphyllobothriidean tapeworms such as *Spirometra* [70]. Cases of *Spirometra mansoni* Cobbold, 1883 have been reported in Costa Rica in 2004, 2022, and 2023 in canine and felid hosts [15, 71], highlighting the need to further investigate the potential role of amphibians and reptiles in local transmission cycles.

The top 10 host species reflect a global pattern, where the dominance of Anura (e.g., *L. melanonotus*, *R. horribilis*) and Squamata (e.g., *Anolis* spp.) as parasite hosts in Mesoamerica mirrors patterns reported from other tropical regions, including South America [45, 46, 72], Africa [50, 73] and South Asia [74, 75]. This can be partially explained by the geographic and climatic conditions of Mesoamerica, which include predominantly tropical climatic conditions, fertile volcanic soils, marked variation in precipitation, pronounced altitudinal gradients (from coastal lowlands to premontane forests), and diverse ecosystems [22]. Host habitat also plays a role in shaping parasite diversity, because it directly determines the scale and frequency of exposure to infective stages present in aquatic, semi-aquatic or terrestrial environments [76]. These factors favor the abundance, persistence, and reproductive success of ectothermic vertebrates such as amphibians and reptiles, contributing to Mesoamerica being one of the world’s most important regions for herpetofaunal biodiversity [77].

Thus, the ectotherm-favorable conditions, combined with habitat heterogeneity, promote high host densities and increase exposure to infective stages, thereby shaping the parasite community structure. In addition, host ecological traits such as broad trophic niches, wide geographic distribution, and larger body size may contribute to the central role of species such as *L. melanonotus* and *R. horribilis* in parasite community structure. Similar patterns have been reported in ecological network studies of amphibian–helminth interactions in Brazil, where leptodactylids and bufonids were identified as important taxa structuring helminth communities [44]. Nevertheless, the apparent dominance of certain host/parasite taxa may be influenced by sampling biases, which tend to disproportionately amplify records of widespread and commonly studied species. Anurans and squamates are generally more abundant, accessible, and easier to detect and sample.

Widespread and abundant species like *R. horribilis* dominate records because they are more easily encountered and sampled across habitats [46, 78]. This may lead to overrepresentation of their parasite records in literature, while understudied taxa (e.g., Caudata, Crocodylia, Gymnophiona) appear rare [45]. This apparent “lower diversity” of parasite records in these groups likely reflects a combination of factors, including difficulties in detection and sampling, limited research and lower abundance in accessible habitats. Conversely, caecilians (Gymnophiona) are poorly known, lacking quantitative data and often cryptic [79]. Similarly, the study of crocodiles often involves logistical and ethical challenges in capturing and sampling, due to their size and conservation status [80]. These gaps highlight the need for targeted and systematic research on understudied host groups. Further analyses incorporating ecological metadata and standardized sampling designs could help untangle true biological patterns from sampling-driven biases.

From a broader macroecological perspective, parasite species richness is largely shaped by local host diversity, meaning that spatial patterns of parasite diversity tend to mirror those documented for their host communities [81]. The observed patterns in this study are shaped by host ecology, sampling efforts, and research focus, rather than absolute parasite richness. Future studies could also benefit from analyzing amphibian and reptile hosts separately, as differences in their evolutionary history, ecology, and biological traits may influence distinct host–parasite association patterns. Moreover, broad taxonomic grouping may mask finer-scale host–parasite interaction patterns, particularly within highly diverse groups such as Squamata. Additional ecological network analyses focusing separately on amphibians and reptilian clades could provide a more detailed understanding of host–parasite associations and interaction structure in Mesoamerican herpetofauna. Integrative approaches combining standardized sampling frameworks, ecological metadata (e.g., habitat characteristics, climatic variables, and trophic interactions), and molecular tools may further help clarify cryptic diversity, transmission pathways, and the ecological and zoonotic implications of host–parasite interactions [53]. Strengthening endogenous research capacity, regional collaborations, and local biological collections will also be essential to improve long-term biodiversity knowledge in the region. Addressing these gaps through systematic sampling, improved taxonomic knowledge, and inclusion of understudied hosts will enhance our understanding of parasite ecology.

Regarding the improvement of taxonomic knowledge of host species, this review also revealed several methodological gaps in the existing literature that merit attention in future studies. The taxonomic classification of the 203 herpetofauna species recorded here has undergone substantial revision over the past two decades, and reconciling historical records with current nomenclature proved challenging in numerous instances. A recurrent obstacle was the lack of precise locality data in earlier publications given that species delimitation in amphibians and reptiles is often critically dependent on geographic provenance, and the absence of detailed collection sites frequently which precluded unambiguous taxonomic assignment of host identities. This underscores the importance of reporting collection localities with sufficient resolution, including geographic coordinates, when possible, as a basic standard in parasitological surveys of herpetofauna.

Equally conspicuous was the near-total absence of ecological context in the studies examined. Very few publications provided even rudimentary environmental information such as elevation, habitat type, vegetation structure, or climatic conditions at the collection site. These variables are well known to influence the distribution and abundance of both amphibians and reptiles and their parasites, and their systematic omission substantially limits the interpretive value of host–parasite records. Future studies would greatly benefit from integrating such ecological descriptors, as this information will be essential for understanding the roles these hosts play within their ecosystems and the ecological dynamics underlying their parasite communities. By strengthening these aspects, future efforts can provide a more accurate and ecologically meaningful picture of parasite communities in Mesoamerican herpetofauna. A substantial portion of the regional helminth fauna remains unexplored and warrants systematic future research.

## Conclusions

This review synthesizes current knowledge on helminth parasites of amphibians and reptiles in Mesoamerica, revealing both ecological patterns and critical research gaps. Nematodes dominate records, and host–parasite associations are concentrated among a few widespread anuran and squamate species. However, the available knowledge remains geographically and taxonomically biased, with most studies originating from Costa Rica and Mexico and largely driven by exogenous efforts, reflecting persistent patterns of parachute science. These asymmetries likely obscure the true diversity, host specificity, and ecological complexity of helminth communities across the region.

Addressing these gaps requires intensified endogenous research, broader sampling in underrepresented regions (e.g., Honduras, El Salvador), of underrepresented species (e.g., Caudata, Crocodylia), and integration of molecular and ecological approaches to detect cryptic diversity. Strengthening local biological collections, promoting equitable international collaborations, and incorporating ecological metadata into future studies will be essential for advancing biodiversity knowledge and improving understanding of parasite transmission dynamics and zoonotic potential within a One Health framework. Overall, Mesoamerican herpetofaunal parasitology remains underexplored. Continued integrative and informed research will be critical for revealing the hidden diversity and ecological roles of helminths in one of the world's most important biodiversity hotspots.

## Supplementary Information

Supplementary Material 1.

## Data Availability

Data are available in Supplementary File 1.

## Abbreviations

USNPC: United States National Parasite Collection
CNHE: National Helminth Collection (Colección Nacional de Helmintos)
CHCR: Helminthological Collection, Laboratory of Helminthology, Faculty of Microbiology, University of Costa Rica
HWML: Harold W. Manter Laboratory of Parasitology, University of Nebraska—Lincoln
COPAFRO: Parasite Collection, El Colegio de la Frontera Sur, Chetumal Unit
IPCAS: Institute of Parasitology, Academy of Sciences of the Czech Republic
DZUT: Department of Zoology, University of Toronto
MHNG: Geneva Natural History Museum, Invertebrate Collection (INVE)
CHIB-UNAM: Helminthological Collection, Institute of Biology, National Autonomous University of Mexico (UNAM)
BMNH: Natural History Museum, London
ECOPA: Colección de Parásitos, ECOSUR-Chetumal
MNHNP: Muséum National d'histoire Naturelle Paris, France
UMMZ: Museum of Zoology, University of Michigan, Ann Arbor, Michigan
